# Fatal Tickborne Phlebovirus Infection in Captive Cheetahs, Japan

**DOI:** 10.3201/eid2409.171667

**Published:** 2018-09

**Authors:** Keita Matsuno, Noriyuki Nonoue, Ayako Noda, Nodoka Kasajima, Keita Noguchi, Ai Takano, Hiroshi Shimoda, Yasuko Orba, Mieko Muramatsu, Yoshihiro Sakoda, Ayato Takada, Shinji Minami, Yumi Une, Shigeru Morikawa, Ken Maeda

**Affiliations:** Hokkaido University, Sapporo, Japan (K. Matsuno, Y. Sakoda, A. Takada);; Hiroshima City Asa Zoological Park, Hiroshima, Japan (N. Nonoue, A. Noda, S. Minami);; Hokkaido University Research Center for Zoonosis Control, Sapporo (N. Kasajima, Y. Orba, M. Muramatsu, A. Takada);; Yamaguchi University, Yamaguchi, Japan (K. Noguchi, A. Takano, H. Shimoda, K. Maeda);; Okayama University of Science, Imabari, Japan (Y. Une);; National Institute of Infectious Diseases, Tokyo, Japan (S. Morikawa)

**Keywords:** phlebovirus, acinonyx, vector-borne infections, viruses, Japan, ticks, cheetah, arthropod-borne infections, zoonoses

## Abstract

Two captive cheetahs from a zoo in Japan died of a severe fever with thrombocytopenia syndrome–like illness. Severe fever with thrombocytopenia syndrome virus, an endemic tickborne phlebovirus, was detected systemically with secretion of infectious viruses into the saliva. These cases highlight the risk for exposure of captive animals to endemic arthropodborne pathogens.

An emerging tickborne virus, severe fever with thrombocytopenia syndrome (SFTS) virus (SFTSV; genus *Phlebovirus*, family *Phenuiviridae* [previously known as family *Bunyaviridae*]) (*1*,*2*), causes severe and often fatal febrile illness in humans, especially in elderly patients. SFTS cases have been identified in East Asia countries (e.g., China, South Korea, and Japan), where the virus also was detected in multiple species of ticks (*3*,*4*) and in domestic and wild animals (*4*,*5*). Ticks and animals play a central role in maintaining the life cycle of SFTSV in the environment and in the occasional transmission of SFTSV to humans. The pathogenesis of SFTSV has been studied in human (*1*,*6*) and animal models using immunocompromised mice that show a lethal SFTS-like illness (*7*,*8*).

In humans, SFTS begins with a high fever, marked thrombocytopenia and leukocytopenia, and a high serum viral load, followed by multiorgan dysfunction, which may be a consequence of systemic inflammatory responses and disseminated intravascular coagulation (*9*,*10*). Gastrointestinal symptoms, such as nausea and vomiting in the early phase and bloody diarrhea in the later phase of the disease, have been frequently reported (*11*). The serum viral load, which can be a prognostic marker associated with a fatal outcome, remains high in fatal cases but decreases in convalescent patients. Here we report 2 fatal SFTS cases in cheetahs in a zoo in the endemic area.

## The Study

In July 2017, anorexia was first recognized in a 7-year-old female cheetah (cheetah 1) in a group of 4 cheetahs sharing the same outside enclosure; she was anesthetized for medical examination on day 3. Laboratory studies showed extremely low leukocyte and low platelet counts and slightly elevated aspartate aminotransferase, alanine aminotransferase (ALT), and total bilirubin levels (Table). The animal was confirmed negative for feline leukemia virus, feline immunodeficiency virus, and feline panleukopenia virus using rapid test kits (Checkman FIV, SNAP FIV/FeLV Combo, Checkman FeLV, and Checkman CPV; Kyoritsu Seiyaku Corporation, Tokyo, Japan). On day 4, cheetah 1 started vomiting with hemorrhage and then died after generalized convulsion. Pathologic analysis identified 4 ulcers in the digestive tract, bleeding in the esophagus, and swollen spleen with white nodules.

**Table:** Hematology and blood chemistry parameters in 2 fatal cases of severe fever with thrombocytopenia syndrome in cheetahs, Japan, 2017

Laboratory value	Normal (± SD)*	Cheetah 1, day 3†	Cheetah 2‡					
			Day 2	Day 3	Day 4	Day 6	Day 7§	
Leukocytes/μL	10,350 (3,500)	1,700	12,500	10,700	6,900	3,900	4,800	
Erythrocytes, × 10^3^ cells/μL	684 (106)	707	845	756	834	722	952	
Platelets, × 10^3^/μL¶	349 (119)	1	12.7	9.1	5.9	0.9	1.3	
Hemoglobin, g/L	12.5 (1.9)	13.9	16.7	15	15.7	14	18.3	
Hematocrit, %	37.9 (5.8)	38.4	58.6	43.1	47.4	39.5	54.4	
Mean cell volume, fL	55.6 (5.5)	54.3	69.3	57	56.8	54.7	57.1	
Aspartate aminotransferase, U/L	52 (35)	161	119	162	145	492	500	
Alanine aminotransferase, U/L	98 (71)	157	412	377	284	501	471	
Creatine phosphokinase, U/L	296 (311)	262	200	915	746	>2,000	>2,000	
Lactate dehydrogenase, U/L	92 (87)	273	203	574	174	684	906	
Total bilirubin, mg/dL	0.3 (0.2)	2.7	0.6	2.9	1.2	5.4	12.3	

*Numbers are obtained from (*12*). †After illness onset. ‡Day 1 was 20 days after cheetah 1 died. §Blood was collected from the carcass. ¶Possible lower platelet counts due to blood collection using heparin.

Slightly abnormal behavior of a 6-year-old male cheetah (cheetah 2) was first observed at 10 and 15 days after the death of cheetah 1. Obvious anorexia in cheetah 2 was recorded at 20 days after cheetah 1 died (hereafter referred to as day 1). Dragging of hind limbs was observed on day 2, and hematologic tests revealed a moderately low platelet count and elevated ALT (Table). A fecal sample was negative for *Helicobacter pylori* antigen, enteric bacteria, and ova-parasite. On day 3, slightly decreased leukocyte count along with continued low platelet count and high liver aspartate aminotransferase levels were detected. On day 4, the gastroscopy test showed erosion and petechiae in the stomach, and a blood-feeding tick was found and removed from the ear. In addition to the low leukocyte and platelet counts, elevated ALT, creatine phosphokinase, and lactate dehydrogenase levels were revealed by laboratory tests on day 6. Cheetah 2 vomited with hemorrhage on days 6 and 7 and died on day 7. Similar to cheetah 1, cheetah 2 had a swollen spleen with white nodules and ulcers in the stomach. No clinical signs were observed in the 2 other cheetahs.

**Figure 1:**
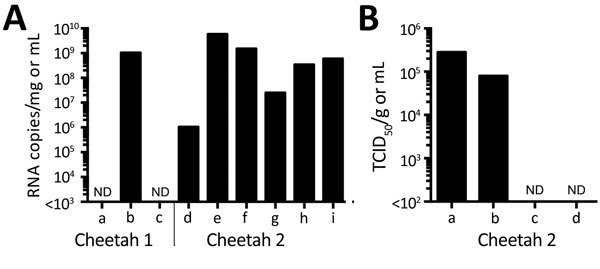
Detection of severe fever with thrombocytopenia syndrome virus (SFTSV) in samples from 2 cheetahs, Japan, 2017. A) RNA was extracted from tissues, plasma, and serum and subjected to quantitative reverse transcription PCR (RT-PCR). The amounts of SFTSV RNA were quantified, with a reference, as RNA copies/mg for tissues and RNA copies/mL for plasma and serum. The mean of duplicate results is shown in the graph. a, plasma; b, popliteal lymph node (left); c, serum; d, brain; e, salivary gland; f, spleen; g, mesentric lymph node; h, popliteal lymph node (left); i, popliteal lymph node (right). B) The TCID_50_ of salivary gland (per mg) and swab specimens (per mL) for cheetah 2 was determined using Huh-7 cells. Virus proteins were detected by an immunofluorescence assay with an anti-SFTSV N monoclonal antibody. a, salivary gland; b, oral swab sample; c, nasal swab sample; d, rectal swab sample. ND, not done; TCID_50_, 50% tissue culture infectious dose.

We performed laboratory tests for virus detection using plasma from cheetah 1 and serum, spleen, and mesenteric lymph node samples from cheetah 2. SFTSV RNA genomes were detected using a quantitative reverse transcription PCR (RT-PCR) targeting the S (small) segment RNA in the spleen and lymph node but not in plasma and serum (Figure 1, panel A). Quantitative RT-PCR showed intensive replication of viral RNA in the popliteal lymph nodes and salivary gland and moderate replication in the brain and spleen. In addition to these tissues, the livers, kidneys, and small intestines of both animals were positive for SFTSV RNAs by conventional RT-PCR. The tissues were negative for flaviviruses, alphaviruses, and canine distemper virus using conventional RT-PCR targeting these viruses.

We isolated infectious viruses using Huh-7 cells and Vero E6 cells from the plasma and popliteal lymph node of cheetah 1 and the spleen, lymph nodes, and brain of cheetah 2 but not from the serum of cheetah 2. Infected cells were clearly stained in an immunofluorescence assay with a monoclonal antibody YG1-7-3-3-4 raised against the recombinant SFTSV nucleoprotein of the Japanese prototype strain YG1. Conditions of the plasma and serum samples may result in negative RT-PCR results in the plasma and negative RT-PCR results and virus isolation in the serum because both samples were collected from animals supposed to cause viremia.

To assess the potential for virus shedding into the secretions of infected animals, infectious SFTSV of the salivary gland, oral, nasal, and rectal swabs from cheetah 2 were titrated using Huh-7 cells. Swab samples were collected from the carcass after freeze and thaw. We detected virus titers of ≈10^5^ 50% tissue culture infectious dose (TCID_50_)/g or TCID_50_/mL in the salivary gland and oral swab samples, respectively; however, virus was not detected in the nasal and rectal swab samples (Figure 1, panel B).

**Figure 2:**
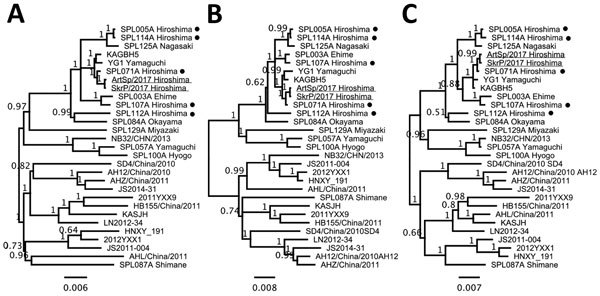
Phylogenetic analyses of severe fever with thrombocytopenia syndrome virus (SFTSV) isolates from 2 cheetahs, Japan, 2017. The phylogenetic trees constructed based on large (A), medium (B), and small (C) segment RNA nucleotide sequences of isolates SkrP/2017 from cheetah 1 and ArtSp/2017 from cheetah 2 (underlined) with representative SFTSV isolates. Isolates from human cases reported in the same prefecture as the zoo are indicated with black dots. The trees were calculated using MrBayes version 3.2.6 (http://mrbayes.sourceforge.net) with the general time reversible plus gamma plus invariate sites substitution model. Numbers beside nodes indicate posterior probabilities. Scale bars indicate nucleotide substitutions per site.

We determined genomic sequences of 2 isolates (SkrP/2017 from the plasma of cheetah 1 and ArtSp/2017 from the spleen of cheetah 2) using MiSeq (Illumina, San Diego, CA, USA) with NEBNext-Ultra RNA Library Prep kit (NEB). De novo assembly on CLC Genomics Workbench (QIAGEN, Hilden, Germany) determined virtually full-length sequences of all 3 RNA segments of 2 SFTSV isolates. We manually edited the termini and remapped virus reads to contigs to define the complete full-length genome sequences (sequences deposited into GenBank under accession nos. LC325234–9). We found only 1 synonymous nucleotide difference on the coding region of the L (large) segment between the 2 isolates. Phylogenetic analyses of 3 RNA segments revealed that both cheetah isolates were clustered together with an SFTSV isolate, SPL071A, which had been reported in a human in the same prefecture as the zoo (Figure 2) (*13*).

## Conclusions

We found a fatal SFTS-like illness of 2 cheetahs naturally infected with an endemic tickborne virus, SFTSV. Disease progression of cheetah 2 was carefully tracked by daily monitoring, providing important clinical information on fatal SFTSV infection in animals. Because the genome sequences of 2 SFTSV isolates were almost identical to each other, and closely related to those of a local isolate from a human case, SFTSV circulating among ticks and wild animals in the area may intrude into the zoo. That 2 cheetahs sharing the same outside enclosure were successively infected with SFTSV within a month of each other and that they had the potential to shed infectious SFTSV into their saliva indicates the virus might have been independently transmitted to 2 cheetahs by ticks; however, the possibility of horizontal transmission through a bite of the animal is undeniable. Further investigation on ticks and animals around the zoo is ongoing. Our study highlights the zoonotic risk for SFTSV infection and the importance of monitoring this endemic arthropodborne disease in zoo animals, as well as livestock, pets, and wildlife.
